# A mHealth intervention to reduce perceived stress in patients with ischemic heart disease: study protocol of the randomized, controlled confirmatory intervention “mStress-IHD” trial

**DOI:** 10.1186/s13063-023-07618-0

**Published:** 2023-09-15

**Authors:** Julia Lortz, Tienush Rassaf, Christoph Jansen, Ramtin Knuschke, Adam Schweda, Lenka Schnaubert, Christos Rammos, Juliane Köberlein-Neu, Eva-Maria Skoda, Martin Teufel, Alexander Bäuerle

**Affiliations:** 1 Department of Cardiology and Vascular Medicine, West-German Heart and Vascular Center Essen, University of Duisburg-Essen, Essen, 45147 Germany; 2 Clinic for Psychosomatic Medicine and Psychotherapy, University of Duisburg-Essen, LVR-University Hospital Essen, Essen, 45147 Germany; 3 Centre for Translational Neuro- and Behavioral Sciences (C-TNBS), University of Duisburg-Essen, Essen, 45147 Germany; 4 Learning Sciences Research Institute, School of Education, University of Nottingham, Nottingham, NG8 1BB UK; 5 Schumpeter School of Business and Economics, Center for Health Economics and Health Services Research, University of Wuppertal, Wuppertal, 42119 Germany

## Abstract

**Background:**

Stress is highly prevalent in patients with ischemic heart disease (IHD) and is associated with lower health-related quality of life and impaired cardiovascular outcome. The importance of stress management is now recognized in recent guidelines for the management of cardiovascular disease. However, effective stress management interventions are not implemented in clinical routine yet. The development of easily disseminated eHealth interventions, particularly mHealth, may offer a cost-effective and scalable solution to this problem. The aim of the proposed trial is to assess the efficiency and cost-effectiveness of the mHealth intervention “mindfulHeart” in terms of reducing stress in patients with IHD.

**Methods and analysis:**

This randomized controlled confirmatory interventional trial with two parallel arms has assessments at six measurement time points: baseline (T0, prior randomization), post-treatment (T1), and four follow-ups at months 1, 3, 6, and 12 after intervention (T2, T3, T4, and T5). We will include patients with confirmed diagnosis of IHD, high-perceived stress, and use of an internet-enabled smartphone. Patients will be randomized into two groups (intervention vs. control). The proposed sample size calculation allocates 128 participants in total. The primary analysis will be performed in the intention-to-treat population, with missing data imputed. An ANCOVA with the outcome at T1, a between-subject factor (intervention vs. control), and the participants’ pre-intervention baseline values as a covariate will be used. Different ANOVAs, regression, and descriptive approaches will be performed for secondary analyses.

**Ethics:**

The Ethics Committee of the Medical Faculty of the University of Duisburg-Essen approved the study (22–11,015-BO).

**Trial registration:**

ClinicalTrials NCT05846334. Release 26.04.2023.

**Supplementary Information:**

The online version contains supplementary material available at 10.1186/s13063-023-07618-0.

## Background

Chronic stress may occur over extended periods from months to years and can result in severe adverse health consequences for individuals. The relationship between chronic stress and cardiovascular diseases continues to be the subject of extensive research [1–5]. Recent large studies have shown that chronic stress is linked to increased risk for cardiovascular diseases (CVD) [2, 6–8], leading to its recognition in current clinical guidelines [9, 10]. Chronic stress has been identified as a risk factor for the development, but also for the progression of CVD, and research has found associations between stress measurements and traditional cardiovascular risk factors [11–14]. Stress is also considered a relevant player in the pathophysiological cascade of coronary atherosclerosis formation (e.g., inflammatory response, endothelial dysfunction, platelet aggregation) [15, 16] until the development of clinical apparent ischemic heart disease (IHD). Measures of stress have been associated with the onset and progression of further cardiovascular disorders, like coronary calcification [17], atrial fibrillation [18], and stroke [19]. In patients with IHD, stress has also been implicated as an acute trigger of myocardial ischemia and infarction, malignant arrhythmias, and sudden cardiac death [20, 21].

Although the body of evidence examining the stress-IHD connection is growing, there continues to be a lack of recognition of this association in clinical practice and of effective and scalable interventions. The sustainable integration of targeted therapy in cardiology practice that involves screening for stress and referral to behavioral therapy or to other stress-reducing interventions (e.g., meditation) would be disarable [22]. Although ongoing group support and concomitant coaching in other lifestyle-related fields such as diet and exercise are conductive to long-term adherence, the establishment of area-wide structured stress management programs is resource-intensive and currently not available.

A potential solution to cost-prohibitive stress reduction programs may lie in the development of easily disseminated eHealth intervention, which can be effective, scalable, and easier to implement in the context of a busy clinical practice [23–25]. The term “eHealth” encompasses a wide range of electronic solutions, such as mobile phones (mHealth) and computers that can enhance and broaden the scope of medical care [26].

Especially mHealth interventions are perceived to offer several advantages that may overcome some of the limitations of face-to-face approaches, including anonymity, 24/7 availability, reduced costs in terms of traveling to courses for both participants and instructors, high scalability, and a low access threshold. Enabling participants to be reached earlier than in classical face-to-face trainings, such interventions, may have the potential to prevent even the onset of more severe chronic stress or mental health problems. The effectiveness of digital interventions for stress reduction was shown in a recent meta-analysis [27].

The link between stress and increased mortality and morbidity in CVD is evident and includes an undeniable reduction in health-related quality of life (HRQoL) [7]. Thus, the search for novel therapeutic strategies seems indicated. Therefore, we aim to evaluate the efficacy and cost-effectiveness of the digital stress management intervention “mindfulHeart” in terms of sustainable stress reduction in the target population.

### Objectives, hypotheses, and other study goals

*The objective* of the “mStress-IHD” trial is to assess the efficacy and cost-effectiveness of the mHealth intervention “mindfulHeart” compared to standard care (control group) in terms of reducing stress in patients with ischemic heart disease (IHD).

*Primary hypothesis*: We expect the intervention to be superior compared to standard care in terms of reducing stress at the end of treatment (T1).

*Secondary hypotheses*: We expect the intervention to be superior to standard care in terms of improving health-related quality of life, functional capacity, self-efficacy, general distress, and stress at follow-up assessment time points (T2-T5). Furthermore, we expect the intervention to be superior to standard care in terms of reducing depression and anxiety symptoms, anger, and perceived stress. We expect the intervention to be superior compared to standard care in terms of cost-effectiveness.

*Other study goals* are to evaluate treatment satisfaction, usability, and perceived usefulness of the mHealth intervention. Furthermore, the study aims to evaluate predictors of usage behavior, time to dropout, and actual usage behavior. Actual usage behavior will be analyzed in terms of its influence on other study outcomes in an explorative approach.

## Methods

This protocol (V.1.0; 15th May 2023) is reported according to the Standard Protocol Items: Recommendations for Interventional Trials checklist (see online Additional file 1) [28]. In case of important protocol modification, it will be reported to the Ethics committee and the trial registration (ClinicalTrials identifier NCT05846334) will be updated.

### Study design

**Fig. 1:**
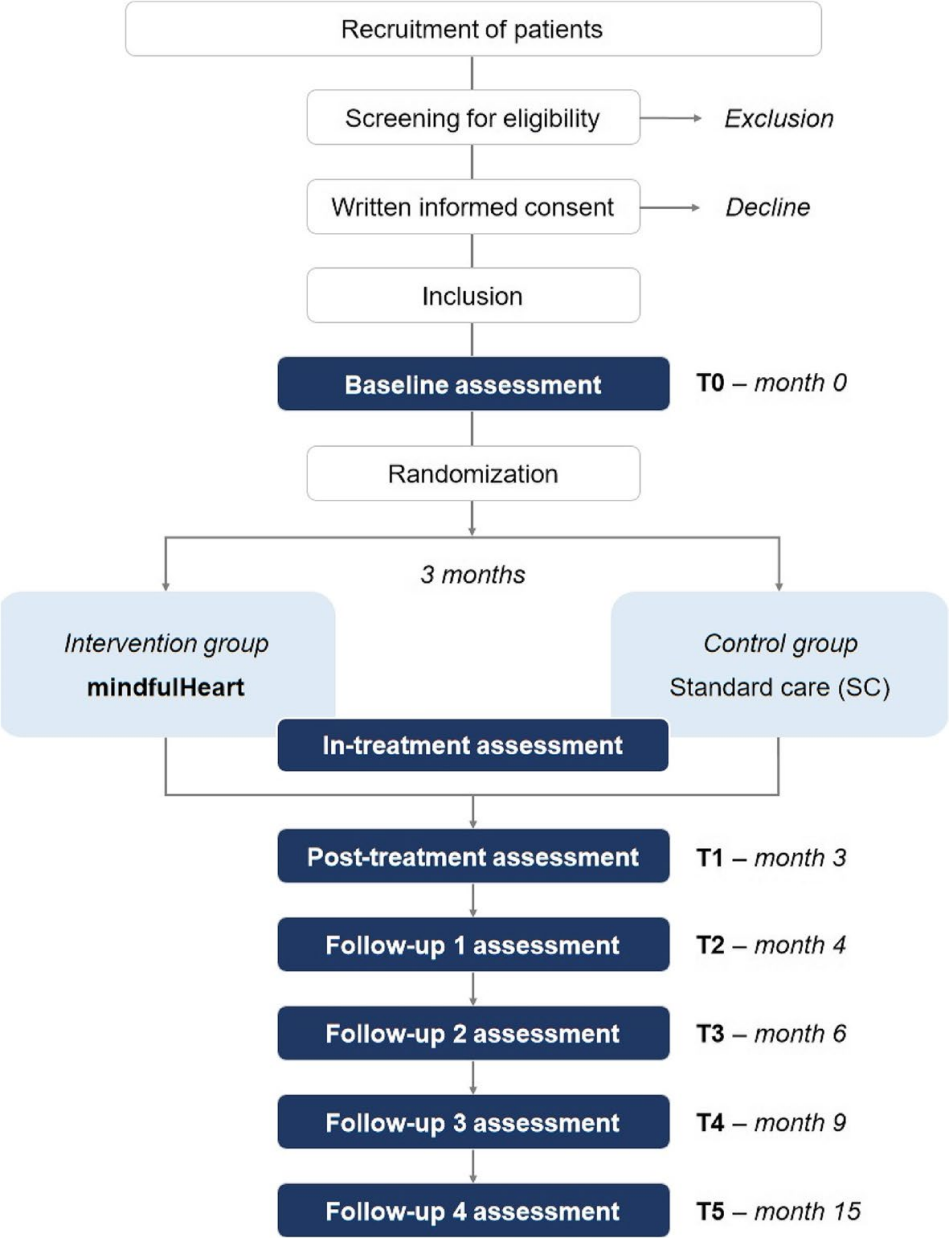
Flow chart of the mStress-IHD trial

The study is a prospective, randomized controlled confirmatory interventional trial with two parallel arms conducted at a single center. The trial includes six distinct measurement time points: the baseline assessment (T0) conducted before randomization, a post-treatment assessment (T1), and four follow-up assessments at 1 month, 3 months, 6 months, and 12 months after the intervention (T2, T3, T4, and T5) as illustrated in Table 1 (assessment schedule) and Fig. 1 (trial flow diagram).

**Table 1:** Overview of the assessment schedule of the mStress-IHD trial

	**Planned assessment time points**				
**Measures**	**Baseline (T0–month 0)**	**During treatment** **(weekly assessment)**	**End of treatment (T1–month 3)**	**Follow-up 1–4 (T2–T5–months 4, 6, 9, 15)**	**Dropout**
**Primary outcome**					
Combined stress measure^a^	x		x	x	x
**Secondary outcomes**					
**Health-related quality of life**					
SF-36	x		x	x	
EQ-5D-5L	x		x	x	
**Angina-related physical function and quality of life**					
SAQ	x		x	x	
**Functional capacity**					
6-min walk test	x		x	x	
**Blood pressure and heart rate**					
Three consecutive measurements at rest	x		x	x	
**Self-efficacy**					
GSES	x		x	x	
**Depression and anxiety symptoms**					
BDI-II	x		x	x	
STAI	x		x	x	
PHQ-4	x	x	x	x	
**Anger**					
PROMIS-Anger	x		x	x	
**Distress**					
GHQ-12	x		x	x	
DT	x	x	x	x	
**Perceived stress**					
PSS	x		x	x	
**Client satisfaction, usability, predictors of usage, and internet-related variables**					
CSQ-I			x		x
SUS			x		x
UTAUT	x				
ETHSA			x		x
Internet-related variables	x				
**Time to drop out and usage behavior**					
Usage behavior via backend		x			
**Demographic and medical characteristics**	x				
**Utilization of healthcare services**	x		x	X^b^	

*BDI-ll* Beck Depression Inventory, *STAI* State-Trait Anxiety Inventory, *PROMIS-Anger*, Patient-Reported Outcomes Measurement Information System-Anger scale, *GHQ* General Health Questionnaire-12 item version; *PSS* Perceived Stress Scale, *SF-36* Short-Form-36 health questionnaire, *EQ-5D-5L* European Quality of Life 5 Dimensions 5 Level Version, *SAQ* Seattle Angina Questionnaire, *GSES* General Self-Efficacy, *DT* Distress Thermometer, *PHQ-4* Patient Health Questionnaire-4, *CSQ-I* Client Satisfaction Questionnaire adapted to Internet-based interventions, *SUS* System Usability Scale, *UTAUT* Unified theory of acceptance and use of technology model, *ETHSA* Evaluation Tool for Healthcare Smartphone Applications, *QALY* cost per additional quality-adjusted life year

^a^The combined stress measure includes Beck Depression Inventory II (BDI-II), State-Trait Anxiety Inventory (STAI), Patient-Reported Outcomes Measurement Information System (PROMIS)–Anger, General Health Questionnaire (GHQ), and Perceived Stress Scale (PSS)

^b^Utilization of health care services will be documented after 6, 9, and 15 months

In addition, continuous assessments will be performed during the experimental intervention to measure the usage behavior of the intervention and other parameters (in-treatment assessment, see Table 1).

Participants who drop out (no login for 4 weeks is considered as a dropout) will be contacted and asked to complete a dropout assessment. Before participating, all patients must provide written informed consent.

### Participant eligibility and recruitment

To be eligible for participation, individuals must have a confirmed diagnosis of IHD. They must be at least 18 years of age, have elevated perceived stress [29] for at least four weeks, own an internet-enabled smartphone and know how to use it, and have provided written informed consent.

Psychosocial stress was assessed by 2 single-item questions relating to stress at work and home. Stress was defined as feeling irritable or filled with anxiety or as having sleeping difficulties as a result of conditions at work or home. The global stress scale combined work and home stress that was graded as (1) never experienced stress, (2) experienced at least 1 period of stress, (3) experienced several periods, and (4) experienced permanent stress. Stress categories were dichotomized into participants with never stressed or at least 1 period of stress (score = 0) and several periods of stress or permanent stress (score = 1), as previously described [29].

Participants who have severe cognitive impairment and/or communication difficulties that may affect their ability to participate in the study have psychiatric or medical conditions that require alternative treatment, and/or do not have private internet access will not be eligible to participate and will be excluded.

Participants will be recruited at the Department of Cardiology and Vascular Medicine, West German Heart and Vascular Center, University Hospital Essen. The study personnel will recruit patients by spreading flyers and posters, as well as contacting patients in the center. Information about the study and the staff involved will also be presented online via social media and the study homepage. Interested patients can obtain more information about the study by phone, in person, or by email.

### Intervention

Randomized patients will be allocated to either the intervention group or the control group. The intervention duration (active study phase) is 3 months.

#### Experimental intervention

The description of the intervention, called “mindfulHeart,” is in accordance to the template for intervention description and replication (TIDieR) [30]. (1) “mindfulHeart” is an interactive, self-guided, and patient-oriented mHealth intervention for the reduction of stress in patients with IHD and includes automated feedback via visualization of changes in patient-reported outcome measures (PROMs). (2) Based on methods of internet-based cognitive behavioral therapy, “mindfulHeart” involves psychoeducational elements, mindfulness-based interventions, and behavioral skills training related to stress management and other health-related topics important for this patient group [31]. The content of the intervention is patient-oriented and need-based, as patients were involved in the development of the intervention [31, 32]. (3) “mindfulHeart” involves different media (e.g., information/education videos, audio-guided mindfulness exercises, or interactive tasks) and is accessible via smartphones. (4) The intervention group will gain access to “mindfulHeart,” consisting of three main components: (a) one weekly themed psycho-cardiological module as well as weekly tasks, (b) measurement to assess the current stress level (via PROMs), and (c) recording, visualization, and the feedback of measurement-history. (a) One psycho-cardiological module per week (in sum 12 modules) on different topics (e.g., stress management) with (psycho-) educational background, skills training, feedback loops to monitor personal progress, and weekly tasks to foster behavior changes in daily lives. At the start of every module, patients are prompt to undertake a brief PROM evaluation, with an emphasis on their current stress level. Subsequently, patients are encouraged to examine their stress course from the past week. Upon completion of each module, patients receive a personalized summary of their progress, a mindfulness exercise regimen, and motivational reminders. Throughout the modules, patients accumulate beneficial techniques and activities in their own skill boxes. Additionally, the intervention entails weekly assignments and mindfulness exercises that should be incorporated into daily routines to promote behavioral modifications. (b) Stress measurements using PROMs will be conducted before each module. Stress measurements using PROMs will be conducted. In addition, patients will be asked in what situation they are (e.g., work, leisure). (c) Records of the PROM measures, including visualization of measurement history in graphs, will give the patient automated feedback. (5) Psychotherapists and physicians (with a specialization in cardiology, as well as psychosomatic medicine?) with a recognized expertise in the field of digital health developed “mindfulHeart.” Furthermore, patients’ representatives were involved in the devolvement of “mindfulHeart” [31]. (6) “mindfulHeart” will be delivered via smartphones. (7) “mindfulHeart” can be used at any time and place, with internet access required. (8) The 12 modules will be unlocked sequentially each week (leading to a total duration of 3 months) and remain at the patient’s free disposal. Each module contains multimodal content and takes about 20–30 min to complete. (9) N/A. (10) N/A. (11) To ensure adherence and fidelity, all patients will receive reminders (e.g., availability of a new module) and motivational messages. Patient-centered UX/UI and gamification elements are integrated to foster adherence to the intervention. (12) N/A.

#### Control condition

The intervention will be compared to standard care. Participants in the control group will have access to the conventional healthcare available in Germany, and their access to other therapeutic and/or medical services during the trial will not be restricted. However, no additional treatment will be offered to the participants allocated to the control group in this trial.

### Outcomes

*The primary outcome* is stress assessed by a combined global stress measure after 3 months (T1, end of treatment). The outcome is based on a published RCT in Circulation by Blumenthal et al. [33]. The combined stress measure is composed of validated instruments, namely the Beck Depression Inventory II (BDI-II), State-Trait Anxiety Inventory (STAI), Patient-Reported Outcomes Measurement Information System (PROMIS)–Anger, General Health Questionnaire (GHQ), and Perceived Stress Scale (PSS). The method involved generating a global measure by assessing each participant’s individual stress measure at baseline and after treatment and ranking them [34]. A mean rank score will then be calculated by averaging the rankings of all stress measures before and after treatment.

*Secondary outcomes for this study include the following:* (a) health-related quality of life (SF-36, EQ-5D-5L), (b) disease-specific effect of angina, (c) changes in functional capacity (6-min walking test), (d) blood pressure and heart rate, (e) self-efficacy, and (f) stress (see primary outcome) over six assessment points. (g) Cost-effectiveness (costs per reduced unit of stress) and cost-utility (costs per additional quality-adjusted life year (QALY)) of the intervention compared to the control group will be assessed in a health economic evaluation.

The components of the primary endpoint ((h) BDI-II, (i) STAI, (j) PROMIS-Anger Scale, (k) GHQ, and (l) PSS) will be used as secondary endpoints. (m) Furthermore, an in-treatment assessment schedule is implemented. We will assess (n) patients’ satisfaction with the intervention (CSQ-I) and usability of the application (SUS), (o) predictors of usage behavior, and (p) time to dropout and actual usage behavior. See below for a detailed description of each secondary outcome.

- *Health-related quality of life:* we will apply two instruments to measure health-related quality of life. Namely the Short Form-36 health questionnaire and the European Quality of Life 5 Dimensions 5 Level survey (EQ-5D-5L) [35, 36]. The SF-36 is an eight-dimensional scale consisting of 36 items. It assesses health-related quality of life based on physical, social, and psychological functioning, role behavior due to physical and psychological functional impairment, physical pain, general health perception, and vitality. The EQ-5D-5L consists of five health-related dimensions that can be assessed at five levels. In addition, the questionnaire contains a visual analog scale for assessing general health.
- *Disease-specific effect of angina on patients’ physical function and quality of life:* We will also include the Seattle Angina Questionnaire (SAQ). The 7-item version of the SAQ is a shortened version of the original 19-item SAQ and has been shown to be highly valid, reliable, and sensitive to clinical change. These ranges of SAQ scores are strongly and independently correlated with the risk of subsequent death, the risk of myocardial infarction, and health care costs [37, 38].
- *Changes in functional capacity:* changes in functional capacity will be recorded using the established 6-min walk test [39].
- *Blood pressure and heart rate:* To evaluate the effect of the intervention on blood pressure and heart rate, these parameters will be assessed with the average of three consecutive measurements at rest. The standard measurement approach will always be performed in the same quiet room at a consistent controlled temperature and after a resting period of at least 20 min. The same examination sequence will be maintained for all study subjects. To obtain reliable measurements, the patients will be asked not to speak and to lie quietly during the entire measurement.
- *Self-efficacy:* To evaluate self-efficacy, we will utilize the Generalized Self-Efficacy Scale (GSES) in its German version [40]. The GSES is a self-administered questionnaire that measures an individual’s optimistic self-beliefs and self-efficacy in dealing with challenging demands and stressful events in life. Each of the 10 items is rated on a 4-point Likert scale.
- *Stress over six assessment points:* stress assessed with the combined global stress measure (see primary outcome) will be measured at each measurement time point and modeled over the whole study period.
- *Cost-effectiveness and cost-utility:* The objective of conducting an economic evaluation is to assess the cost-effectiveness of the intervention from an economic viewpoint. This evaluation will involve a cost-effectiveness analysis and a cost-utility analysis. To determine the cost-utility, quality-adjusted life years will be calculated based on the EQ-5D-5L questionnaire. The resource utilization will be evaluated by means of a questionnaire and will be measured in monetary units using the established standards of health economics. Furthermore, the costs per reduced unit of stress are assessed and compared between the intervention group and control group.
- *Depression symptoms (BDI-II):* The BDI-II is a self-report questionnaire comprising 21 items that assess depression severity. Its reliability and validity have been established through various studies among diverse populations and cultural backgrounds. Higher scores indicate increased depression symptoms [41].
- *Anxiety symptoms (STAI):* The STAI is the most authoritative tool for assessing anxiety in adults, precisely distinguishing between transient “state anxiety” and persistent “trait anxiety.” The STAI measures anxiety with 20 items and has a range of 20 to 80. A higher score indicates more pronounced anxiety [42].
- *PROMIS-Anger Scale*: The PROMIS Anger scale comprises eight items that evaluate various aspects of anger. Scores on the scale range from 8 to 40, with higher scores indicating greater levels of anger [43].
- *GHQ:* The GHQ assesses general distress and consists of 12 items. Respondents’ scores range from 0 to 36, where higher scores correspond to increased distress [44].
- *PSS:* The PSS-10 item version is a short tool for the assessment of how individuals perceive stress in their lives. The values range from 0 to 40. Higher values indicate higher perceived stress [45].
- *In-treatment assessments:* At the start of each intervention module (weekly), the following assessment instruments will be applied: Distress Thermometer (DT), Patient Health Questionnaire-4 (PHQ-4), and self-generated measures to assess coping skills and self-efficacy [46, 47].
- *Treatment satisfaction und usability:* The adapted German version of the Client Satisfaction Questionnaire for Internet-based interventions (CSQ-I) is an 8-item assessment instrument that evaluates participants’ overall satisfaction with the intervention [48]. Responses are measured on a 4-point Likert scale and combined to an overall satisfaction value. Additionally, the System Usability Scale is used to assess the usability of the intervention, which is a 10-item questionnaire with each item rated on a 5-point Likert scale combined to an overall score [49]. To determine the perceived usefulness of our healthcare smartphone app, we will also use a healthcare smartphone app evaluation survey [50]. Responses are measured on a 4-point Likert scale and combined for an overall score.
- *Predictors of usage behavior:* Assessing the predictors of actual usage behavior is crucial as interventions can only benefit patients who use them. Therefore, it is important to evaluate the predictors of uptake for the intervention. In order to do so, the Unified Theory of Acceptance and Use of Technology will be applied [51]. Additionally, internet-related scales (internet anxiety, digital confidence, duration of internet usage, eHealth Literacy) will be assessed [52].
- *Time to drop out and actual usage behavior:* The determination of dropout and usage behavior will be based on various factors such as the number and type of modules completed, the duration of usage per day, the type and number of modules initiated, the time elapsed since the last login, the frequency of logins, the frequency of each module, the time spent on each module, the percentage of each module completed, the type and quantity of videos and audios initiated and completed. Data will be captured through the backend of the intervention.

Medical data (e.g., prior myocardial infarction, prior bypass surgery) and sociodemographic data (e.g., sex, age, and education) will be assessed at baseline (T0). Relevant changes in health status are continuously monitored and documented.

### Trial procedure and timeline

Figure 1 and Table 1 provide an overview of the trial flow and assessment schedule, respectively. The study duration is 15 months, which includes 3 months of the intervention period (active phase) for the participants. The last follow-up visit will take place 12 months after the intervention. Assessments will be conducted at the Department of Cardiology and Vascular Medicine, West German Heart and Vascular Center, University Hospital Essen, Germany.

Interested patients will be screened for eligibility and receive further information about the study conditions, data storage, and data safety (oral and written). The study team at the respective clinic will conduct a baseline diagnostic assessment to determine eligibility. If the patient meets the inclusion criteria and does not have any exclusion criteria and provides written informed consent, they will be included in the study.

After completing the baseline assessment, the patient will be randomly assigned to either the intervention group or control group. Patients will be notified of their group allocation and will receive information on how to access the intervention (intervention group). Participants allocated to the control group will receive information about the further steps (assessment schedule). At the end of the 3-month experimental or control intervention period, patients will complete a post-treatment diagnostic assessment and four follow-up diagnostic assessments at one, 3, 6, and 12 months after the intervention.

If a patient drops out during the intervention (i.e., no login for 4 weeks), they will receive an e-mail containing a link to a short questionnaire that assesses their reasons for dropping out.

### Sample size calculation

The sample size calculation was performed using the R-package “Superpower” [53]. The primary hypothesis is that the intervention is superior in terms of reducing stress compared to standard care (control group) at T1. To assess the efficacy of the intervention, an ANCOVA with the primary outcome variable at T1 as the criterion, the group variable (intervention vs. control group) as a between-subject factor, and the individual baseline levels at T0 as a covariate will be performed. A subsequent mixed ANOVA with the respective measurements (T0–T5) as a within-subject factor and the group variable (intervention vs. control group) as a between-subject factor will then serve to explore the intervention’s effect at follow-up. For the sample size calculation, a preferred test power of 1-ß = 0.80 was chosen. We assume a moderate effect size of *d* = 0.5 for the baseline-adjusted comparison between the intervention and control group at T1. Furthermore, and based on previous studies [54], we expected the correlation between repeated measures to be *r* = 0.5. According to the power analyses, an effective sample size of *n* = 98 (i.e., 49 participants per group) would yield a statistical power of 80.34 for the above mentioned ANCOVA analysis. With the same amount of participants, we reach a power of 95.8 for the above-mentioned mixed ANOVA (given a rather conservative assumption of the sole difference between the intervention and control group to occur at T1). Based on existing literature regarding eHealth trials [55], we assume a dropout rate of 30%. Based on the preliminary work related to this study protocol [31, 32] and recommended measures to increase usability, especially user experience [55], a dropout rate in the lower level was assumed. Consequently, 128 participants (64 per group) are needed.

### Randomization and blinding

Balanced block randomization (1:1 randomization) will be applied, using a standard computer algorithm. No stratification will be applied. It is assumed that the randomization process will result in a balance of the prognostic factors. The study personnel will communicate the result of the allocation to the participants after enrollment and randomization process.

The blinding process will include almost all stakeholders within the treatment process. Treating physicians, just like assessors and statisticians (primary analysis) will be blinded to group allocation to ensure objective analyses. Only blinding for patients will be performed due to the knowledge of each individual about the treatment with or without an app. Demasking for physicians, assessors, or statisticians is not planned.

### Data management, data storage, and dissemination policy

To ensure participant confidentiality, their data will be pseudonymized. All standard operating procedures will adhere to legal requirements, as outlined in the data management plan. Both the study team and the patients themselves will enter data electronically. Paper-based data will be transferred to the electronic database. Following Good Clinical Practice guidelines, important trial data (including signed informed consent forms, patient identification lists, and original clinical findings) will be archived for 10 years after the trial’s completion or termination. Patient documents will be stored in line with hospital regulations. Stored data during and after the trial will only be accessible to authorized staff.

We will adhere to Good Clinical Practice guidelines and follow the data-sharing statements outlined in The New England Journal of Medicine [56, 57]. Once the major results of the trial are published, the collected data will be available upon reasonable request in an anonymous format. In addition, we will store and make available the statistical analysis plan and other relevant documents upon request. Access to the data storage will be limited to authorized personnel only. Patient consent forms will include a section covering the aforementioned aspects of data storage and sharing.

Our approach to disseminating the trial’s progress and outcomes will include several measures. First, we will establish a project homepage that will provide regular updates to the public about the trial’s development, opportunities for participation (such as workshops and conferences), and significant findings. This website will remain active even after the recruitment is closed, serving as a central point of access for future research and results dissemination. We will also publish the main trial results open-access in a peer-reviewed journal and make them publicly available in the clinical trial registry. Additionally, we will present the findings at conferences and communicate scientific results in lay language through press releases, social media, and patient forums. To ensure the results reach the affected population and the public, we will collaborate with patient representatives and patient organizations to develop targeted, patient-oriented information campaigns.

### Statistical methods

The primary goal of this trial is to assess the efficacy of the intervention compared to the control group in terms of reducing stress directly after the intervention. For this purpose, an analysis of covariance (ANCOVA) with the outcome at T1, a between-subject factor (intervention group vs. control group), as well as the participants’ pre-intervention baseline values (T0) as a covariate will be used. The primary analysis will be performed in the intention-to-treat population, with missing data imputed in the case of dropout. Imputation will be calculated using the SPSS multiple imputation module with “monotone missing pattern” (as we will use complete data for sex, age, and baseline data of primary and secondary outcomes). The number of imputations will be set to 3000, and the seed will be set to the date of the analysis (ddmmyy). Interim analyses are not planned.

Subsequent mixed ANOVAs with a between-subject factor coding the group membership (intervention vs. control group) and a within-subject factor for the respective measurement (T0-T5) will serve to assess if the effect of the intervention is maintained at follow-up. Further clinical endpoints will be analyzed in a similar fashion. If, in any of the above-mentioned analyses, we find severe violations of normality or homoscedasticity, robust procedures (e.g., robust regression analyses, generalized estimating equations, or robust mixed linear models) will be used, instead. In case of violation of the sphericity assumption in the mixed ANOVA, appropriate corrections will be used (Huynh-Fieldt or Greenhouse–Geisser).

Treatment satisfaction, usability, and usage behavior will be analyzed descriptively. Furthermore, *t*-tests (or, in the case of a violation of the normality assumption, the Mann–Whitney *U*-test) and, for categorical variables, *χ* ^2^ tests (or, for variables with small cell sizes, Fisher’s exact tests) will be used to account for unlikely but possible differences in demographic and baseline parameters between the two groups. In addition, different groups (e.g., dropouts; group completing the intervention; group benefiting from the intervention) will be analyzed using analyses of variance and *χ* ^2^ tests to determine whether they differ from each other in terms of sociodemographic and clinical data.

Furthermore, exploratory analyses including usage behavior and clinical outcome parameters (all assessments, including in-treatment assessments) will be conducted using, for example, different types of regression analyses. Time to dropout will be examined as a secondary endpoint, utilizing both the Kaplan–Meier estimate and the Cox proportional hazards regression.

Additionally, separate tabulations and line listings of adverse events and severe adverse events will be generated to thoroughly analyze safety.

To evaluate the cost-effectiveness of the intervention, the incremental cost-effectiveness ratio (ICER) and incremental cost-utility ratio (ICUR) will be calculated as the ratio of the mean cost difference and the mean difference in health effects (measured in terms of either stress (primary outcome) or QALY) between study groups during the follow-up period. The analysis will also consider the uncertainty of results.

### Ethical aspects

In our opinion, the trial does not raise any ethical concerns. We are not aware of any specific risks or disadvantages that may affect patients during the trial, and we do not anticipate any specific adverse or serious adverse events caused by the intervention or due to participating in the trial. However, in the event of unexpected adverse or serious adverse events, they will be thoroughly documented and reported. Furthermore, dropouts will be assessed and documented. Throughout the trial, patients will have access to telephone/video consultation or face-to-face contact with a member of the study team as well as a senior cardiologist as needed.

The Ethics Committee of the Medical Faculty of the University of Duisburg-Essen have approved the conductance of the study (22–11,015-BO). The trial will be conducted in accordance with the tenets of the Declaration of Helsinki and the guidelines of Good Clinical Practice.

In order to participate, written informed consent after receiving both oral and written information, is mandatory. Informed consent will be obtained prior to randomization. However, participants have the right to withdraw from the study at any time without any negative consequences.

### Patient and public involvement

Patients were involved in developing the intervention and in planning the proposed trial. The intervention is based on a user-centered design approach [31]. Furthermore, we have assessed the acceptance and potential drivers and barriers to use the presented intervention [32]. Patient representatives were involved in the compilation of patient-friendly summaries and consent forms. Furthermore, we will work closely with patient representatives and self-help organizations in terms of dissemination of results among the target group. The final case report forms were tested regarding comprehensibility, illustration, and duration of processing by the patient representatives.

The public is informed by publication of the results in scientific journals, at professional congresses as well as publication of the results in lay language on the social media channels and the study website.

## Discussion

The mStress-IHD study is a randomized-controlled trial to investigate whether the digital stress management intervention “mindfulHeart” is capable of reducing stress effectively, sustainable, and cost-effective in patients with IHD. If the level of stress reduction is effective, as defined by a composite stress measure [33], the planned trial will be the first to show that the perceived stress level can be influenced by a mHealth intervention in this vulnerable patient population.

Patients with IHD experience an enormous burden with their daily stress load, composed of psychosocial stressors and additional disease-specific components, that lacks adequate treatment options in general clinical practice [58, 59]. The general access to psychosomatic intervention including stress management programs is limited in access to nonexistent in standard care, although current guidelines make a clear recommendation to implement stress counseling as an important component of secondary prevention [9, 10]. However, the reality reflects a lack of contact points, full appointment calendars, and interdisciplinary collaboration that needs to be expanded [60, 61]. It is important to know whether mHealth intervention bears the potential to become an integrative part of the treatment phase of patients with IHD to improve quality of life and at best cardiovascular prognosis, too.

There are some aspects worth mentioning that could limit the significance of the planned study. Firstly, since the participation is voluntary, severely distressed patients might escape the screening due to overwhelming by the actual events for participation. Secondly, the blinding of participants is only unilateral due to the parallel study design, which is a common limitation in mHealth intervention studies. Thirdly, we will only recruit patients at a single center. Future studies should take these limitations into account.

The mStress-IHD trial has several strengths; foremost its external validity, since IHD patients are consecutively recruited from the cardiology and vascular medicine department of a tertiary center with a wide rural and urban catchment area. In addition, the assessed psychometric and biomedical outcome variables and covariates are well-established and cutting-edge measures in behavioral cardiology studies. To this extent, they may provide important novel insight into the prevention of chronically elevated stress in the context of IHD and its link with psychosocial functioning and cardiometabolic risk, while also allowing us to identify potential moderating and/or confounding factors of these relationships. Finally, the mStress-IHD study might extend our knowledge about the benefits and limits of mHealth stress management programs for IHD patients with a potential to reduce the burden on the health care system.

In conclusion, the mStress-IHD trial might contribute to the knowledge of mHealth interventions in general and especially for patients affected by IHD. This knowledge might be important to implement evidence-based digital intervention for patients to overcome existing barriers in everyday healthcare and support patients’ well-being.

### Supplementary Information

**Additional file 1:** **Table 1.** SPIRIT Checklist.

## Abbreviations

CVD: Cardiovascular disease
IHD: Ischemic heart disease
HRQoL: Health-related quality of life
TIDieR: Template for intervention description and replication
PROMs: Patient-reported outcome measures
BDI-II: Beck Depression Inventory II
STAI: State-Trait Anxiety Inventory
PROMIS: Patient-Reported Outcomes Measurement Information System
GHQ: General Health Questionnaire
PSS: Perceived Stress Scale
QALY: Quality-adjusted life year
EQ-5D-5L: European Quality of Life 5 Dimensions 5 Level
SAQ: Seattle Angina Questionnaire
GSES: Generalized Self-Efficacy Scale
DT: Distress Thermometer
PHQ-4: Patient Health Questionnaire-4
CSQ-I: Client Satisfaction Questionnaire for Internet-based interventions
ANCOVA: Analysis of covariance
ICER: Incremental cost-effectiveness ratio
ICUR: Incremental cost-utility ratio
